# Retinoids, vitamin D, invasion, and metastasis

**DOI:** 10.3747/co.v13i6.120

**Published:** 2006-12

**Authors:** E.J. Tokar, R.J. Ablin, M.M. Webber

**Affiliations:** * Inorganic Carcinogenesis Section, National Cancer Institute at the National Institute of Environmental Health Sciences, Research Triangle Park, North Carolina, U.S.A; † Department of Immunobiology, University of Arizona College of Medicine, and the Arizona Cancer Center, Tucson, Arizona, U.S.A.; ‡ Departments of Zoology and Medicine, Michigan State University, East Lansing, Michigan, U.S.A

**Keywords:** Chemoprevention, invasion, metastasis, prostatic intraepithelial neoplasia, prostate cancer, retinoids, vitamin D

## INTRODUCTION

Prostate cancer has a long latency period: 20 to 30 years or more. During this time, cells acquire malignant characteristics in the multistep process of carcinogenesis. Prostatic intraepithelial neoplasia (pin), the precursor of cancer, which may progress to invasive cancer, has been observed in men in their twenties and thirties; however, the cancer is not generally manifested until after age 60. It may therefore be possible to block the progression of pin not only to high-grade pin, but also to early carcinoma, by using chemopreventive agents during the long latency period. Thus, pin provides an extraordinary opportunity for prostate cancer prevention. In addition, secondary and tertiary chemoprevention implies intervention in the multistep process of carcinogenesis not only to reverse or arrest premalignant lesions, but to also prevent invasion and metastasis.

## BACKGROUND

To invade and produce metastasis, metastatic cells must exhibit a number of characteristics, including changes in cell adhesion, vimentin expression (associated with increased cell motility), and ability to degrade the extracellular matrix (ecm) by secretion of proteinases. Cells with some or most of these characteristics may exist in the heterogeneous cell population of very early cancer.

In addition, before metastasis, the primary tumour must also create neoangiogenesis. The leakiness of tumour neovasculature facilitates entry (intravasation) of cancer cells into the bloodstream. The secretion of proteinases is necessary not only for cancer cells to degrade the basement membrane and invade the underlying tissue, but also for angiogenesis and for the entry of cancer cells into the blood vessels at the primary tumour site and for their exit (extravasation) from blood vessels in distant organs to establish metastases.

Many natural and synthetic agents with the ability to inhibit carcinogenesis, invasion, and tumour progression have been identified. Vitamin A, vitamin D, and their natural and synthetic derivatives are known inhibitors of carcinogenesis in a wide variety of tissues because they exert multiple effects on preneoplastic cells such as those in pin and on carcinoma cells. Many retinoids have the ability to inhibit cell proliferation, induce differentiation, and reduce proteinase expression, motility, and invasion by cancer cells^1^.

Webber and Tokar recently showed that cholecalciferol (vitamin D_3_), a precursor for the active hormone calcitriol, has the ability to inhibit changes associated with malignant transformation^2^,^3^. They further demonstrated the presence of 25-hydroxylase (CYP27A1), an enzyme required for the synthesis of calcitriol, in human prostate epithelial cells. Those observations—and the fact that cholecalciferol is non-toxic and readily available at low cost—make cholecalciferol an important agent for prostate cancer prevention and treatment.

## EFFECTS OF RETINOIDS AND VITAMIN D

Retinoids and vitamin D may have many different modes of action. Natural and synthetic derivatives of vitamin A (retinoids), such as all-*trans-*retinoic acid (atra), *N-*(4-hydroxyphenyl)retinamide (4-hpr), and derivatives of vitamin D are being tested and used for their preventive and therapeutic activity for prostate and other cancers.

Vitamins A and D play an important role in normal epithelial cell proliferation and differentiation. Their ability to inhibit carcinogenesis, cancer cell growth, invasion, and metastasis has been the focus of studies by Webber and colleagues^1^–^5^. The objective of those studies has been to examine, *in vitro,* the ability of retinoids and vitamin D alone or in combination to block or reverse the changes associated with malignant transformation, invasion, and tumour progression, and to examine their mechanisms of action.

Epidemiologic studies show an inverse relationship between serum levels of vitamins A and D and prostate cancer risk, suggesting that these vitamins have anticancer properties. By themselves, atra, 4-hpr, and cholecalciferol inhibit anchorage-dependent and -independent growth of prostate cancer cells^1^,^3^,^4^; when used in combination, 4-hpr and cholecalciferol have synergistic effects.

Another characteristic of malignant cells is a decrease in or loss of the ability to undergo apoptosis. Restoration of the ability to undergo apoptosis in pre-malignant and cancer cells is considered to be a valuable property of chemopreventive and chemotherapeutic agents. Retinoids and vitamin D can induce apoptosis in prostate cancer cells.

Invasion is a critical step in the metastatic cascade. Cancer cells secrete a number of proteinases, including urokinase-type plasminogen activator (upa) and matrix metalloproteinases (mmps), which have the ability to degrade basement membrane and other ecm proteins and to facilitate invasion and metastasis. A direct correlation between proteinase activity and metastatic potential has been observed. This suggests that blocking or inhibiting the action of mmps and upa could have preventive effects by inhibiting the early stages of carcinogenesis and invasion and metastasis. Increased levels of mmp-2 have been observed in pin and with increasing Gleason score in prostate cancer, suggesting an inverse relationship between differentiation and levels of mmp-2. Because tumour angiogenesis is a necessary step in the metastatic cascade, it is important to note that mmps are upregulated in proliferating endothelial cells and contribute to^2^,^3^ and 4-tumour angiogenesis. Cholecalciferol hpr both induce a marked decrease in mmp-2 and mmp-9 activity (unpublished data).

Webber *et al.* have also shown that atra markedly reduces the activity of upa secreted by human prostate cancer cells^4^,^5^. A correlation between upa secretion, malignant transformation, invasion, meta-static potential, and cancer aggressiveness has been documented. Urokinase-type plasminogen activator occupies a place at the apex of the proteolytic cascade and initiates the degradation process. Subsequently, collagenases are recruited after activation of pro-collagenases by plasmin formed by the activation of plasminogen by upa. And not only plasmin, but upa by itself can degrade ecm proteins 5. However, treatment of prostate cancer cells with retinoids reduces upa activity, ecm degradation, and invasion^1^,^4^,^5^. These findings may explain one mechanism by which retinoids inhibit invasion and thus block progression of pin to invasive cancer and metastasis.

Naturally occurring intrinsic inhibitors play an important role in proteinase inhibition. These include tissue inhibitors of mmps (timps) and plasminogen-activator inhibitors (pais), which regulate proteinase activity. During normal tissue remodelling, homeostasis is maintained between proteinases and their inhibitors; but, in cancer cells, this homeostasis is lost. Agents that can induce an increase in the levels of timps and pais are important in inhibiting invasion and metastasis. Because many retinoids and vitamin D analogs can inhibit invasion as well as angiogenesis, one possible explanation of these effects is that they increase production of transforming growth factor beta (tgfβ)—a potent inhibitor of epithelial cell proliferation—in prostate epithelial cells.

Webber *et al.* have shown that tgfβ inhibits the growth of the malignant RWPE2 human prostate epithelial cells^6^. In addition, tgfβ can increase the synthesis of ecm proteins, timps, and pais, thus reducing the potential for invasion and metastasis by maintaining basement membrane integrity. Retinoids stimulate ecm production, alter cell adhesion properties, modulate tgfβ production, and inhibit angiogenesis. Because degradation of endothelial ecm is necessary for angiogenesis, upa secreted by cancer cells will promote angiogenesis in very small tumours and will enhance invasion and metastasis. Cholecalciferol, atra, and 4-hpr have the ability to reduce net extracellular proteolytic activity and thus reduce ecm degradation and invasion—and consequently metastasis^1^–^5^.

## INVASION AND METASTASIS

To be able to invade and metastasize, cancer cells must be motile. Changes occur in the expression of intermediate filament proteins (ifps) during malignant transformation. Cytokeratins are the normal ifps expressed in epithelial cells; during carcinogenesis and tumour progression, co-expression and increased expression of vimentin has been observed. Vimentin is a growth-regulated gene often expressed in rapidly proliferating carcinoma cells. Vimentin expression is associated with a de-differentiated phenotype, increased ability for motility and invasion^1^,^3^, drug resistance, and poor clinical prognosis. In the prostate, vimentin expression has been observed in highly metastatic tumours^1^.

Cell characteristics that enhance the invasive ability of cancer cells are also likely to enhance their meta-static ability. Agents that downregulate vimentin expression and other cellular changes associated with invasive ability are therefore useful in the treatment of metastatic disease. Retinoids and cholecalciferol have been shown to induce cell differentiation and to markedly reduce vimentin expression^1^,^3^. It is therefore reasonable to suggest that agents that reduce vimentin expression may have chemopreventive potential because of their ability to reduce cancer cell motility.

## MECHANISMS OF ACTION

Having observed the effects of retinoids and vitamin D, the next step is to examine their mechanisms of action. Retinoids and vitamin D exert their effects primarily through their receptors, which are members of the nuclear receptor super-family of ligand-activated transcription factors. Retinoid action is mediated by two families of receptors, the retinoic acid receptors (rars) and the retinoid-X receptors (rxrs), each of which have three subtypes—α, β, and γ. Vitamin D effects are mediated by the vitamin D receptor (vdr) after heterodimerization primarily with rxrs (although vdr can also form heterodimers with rars). The action of 4-hpr is mediated by rxr/rar heterodimers. The dimerization between vdrs and rxrs and rars indicates an interaction between vitamin D and the retinoid signalling pathways, which may in part explain the synergistic effects. Treatment of prostate cancer cells with cholecalciferol, 4-hpr, or atra results in upregulation of vdr and certain subtypes of rxrs and rars. The upregulation of the various subtypes depends on the agent being used.

## CONCLUSIONS

These observations suggest that non-toxic agents such as retinoids and vitamin D, which have the ability to inhibit growth, induce differentiation, inhibit vimentin expression, reduce cell motility, inhibit upa and mmp activity, and inhibit invasion and angiogenesis, are potential cancer prevention and intervention agents. Based on the upregulation of nuclear receptors, the effects of these agents are primarily receptor-mediated. These findings are significant, because vitamin D and retinoids are both important chemopreventive agents and their combined use permits a significant increase in drug efficacy at lower doses, thus eliminating the risk of toxicity^7^. These *in vitro* studies should be translated into *in vivo* experiments. For *in vivo* studies, Webber and colleagues have developed cell lines derived from RWPE1 cells^6^,^9^–^11^ by transformation with Ki-*ras* oncogene^6^,^8^ or the chemical carcinogen *N-*methyl-*N*-nitrosourea^9^. This family of cell lines, all with the same lineage, show a progressive increase in malignancy and metastatic potential^11^. These cell lines provide useful *in vivo* models to investigate the efficacy of agents for chemoprevention and treatment before human trials for the prevention and treatment of meta-static prostate cancer are conducted.
